# Combined effects of heat and rhamnolipids against *Listeria monocytogenes*: Influence of water activity and pH

**DOI:** 10.1007/s11274-026-05352-8

**Published:** 2026-10-16

**Authors:** Gustavo Vasconcelos Gomes, Marcia Nitschke

**Affiliations:** 1 Interunits Graduate Program in Bioengineering (EESC/FMRP/IQSC), University of São Paulo, Trabalhador São-carlense Av., 400, São Carlos, São Paulo, 13566-590 Brazil; 2 São Carlos Institute of Chemistry (IQSC), University of São Paulo, Trabalhador São-carlense Av., 400, São Carlos, 13566-590 SP Brazil

**Keywords:** Biosurfactants, Hurdle technology, Rhamnolipids, Thermal inactivation

## Abstract

Foodborne diseases remain a major public health concern, with *Listeria monocytogenes* being one of the most significant foodborne pathogens due to its high mortality rate and persistence in food-processing environments. This study evaluated the combined effects of heat, pH, and water activity on the antimicrobial activity of rhamnolipids (RL) against *L. monocytogenes* within the framework of hurdle technology. Treatments were performed at 62 °C for 10 min under different pH and water activity (a_w_) conditions adjusted with NaCl or sucrose. Increasing NaCl concentrations reduced the efficacy of heat treatment alone; however, its combination with RL produced a synergistic effect, achieving reductions exceeding 5 log CFU/mL in low-a_w_ media containing NaCl. In sucrose-containing media, the combined treatment reduced viable populations by 4–5 log CFU/mL across all pH and a_w_ conditions evaluated. The hurdles also showed potential as a sanitizer formulation against *L. monocytogenes* attached to stainless steel surfaces, while scanning electron microscopy revealed extensive structural damage to treated cells. The combination of moderate heat and rhamnolipids exhibited synergistic antimicrobial activity under a range of pH and water activity conditions, representing a prospective strategy for controlling *L. monocytogenes*, particularly in low-water-activity foods with high salt or sugar contents.

## Introduction

Unsafe food is estimated to cause 866 million illnesses and 1.5 million deaths worldwide each year, resulting in substantial economic losses (WHO 2026). Among foodborne pathogens, *Listeria monocytogenes* is of particular concern because it causes listeriosis, a severe disease associated with high hospitalization and mortality rates (dos Santos et al. 2021). Its ability to tolerate a wide range of environmental conditions, including variations in pH, temperature, and water activity (a_w_), together with its capacity to form biofilms and persist in food-processing environments, contributes to its survival and dissemination throughout the food chain (Bucur et al. 2018; Pang et al. 2019; Osek et al. 2022; dos Santos et al. 2021). Therefore, effective approaches for controlling *L. monocytogenes* while maintaining food quality remain an important challenge for the food industry.

Biosurfactants (BS) are microbial surface-active compounds that have attracted increasing interest as alternatives to conventional synthetic surfactants because of their biodegradability, low toxicity, and potential production from renewable substrates (Mulligan et al. 2014). Rhamnolipids (RL), glycolipid BS produced mainly by species of the genus *Pseudomonas*, exhibit surfactant, emulsifying, and antimicrobial properties against several foodborne microorganisms (Nitschke and Silva 2018). Their antimicrobial activity may be influenced by environmental conditions (Passos and Nitschke 2024a, b), suggesting that combining RL with other physical factors could improve their effectiveness. Such an approach is consistent with the hurdle technology concept, in which combinations of preservation factors are used to enhance microbial inactivation while potentially reducing the intensity of individual treatments (Leinster 2000).

Although heat treatment is widely used to control microorganisms in foods, intensive thermal processing can adversely affect nutritional and sensory quality (Abel et al. 2022). Combining moderate heat with complementary antimicrobial factors offers an alternative to more intensive thermal treatments; however, the efficacy of these combinations depends on environmental conditions, including pH and water activity ( a_w_). In particular, reducing a_w_ through the addition of solutes such as NaCl or sucrose influences microbial stress responses and thermal resistance (Beuchat et al. 2013; Burgess et al. 2016). These factors are relevant to *L. monocytogenes*, which can tolerate osmotic stress and survive under reduced a_w_ conditions (Osek et al. 2022).

Previous studies from our research group have shown that environmental conditions influence the antimicrobial activity of RL. Ferreira et al. (2019) reported greater RL activity against Gram-positive foodborne pathogens at pH values between 5.0 and 6.0. More recently, the presence of NaCl or sucrose was shown to enhance the antimicrobial activity of RL against *L. monocytogenes* (Passos and Nitschke 2024a, b). These findings suggest that pH and solute may modulate RL activity and provide a basis for combining RL with additional preservation strategies.

However, limited information is available on the combined effect of RL and mild heat under different pH and a_w_ conditions. Lenaerts et al. (2023) reported enhanced thermal inactivation of *L. monocytogenes* Scott A at 60 °C when RL was added in reduced-a_w_ buffer systems containing glycerol and NaCl (a_w_ = 0.92). Nevertheless, the effects of combining RL and moderate heat across different pH and a_w_ conditions, particularly using different food-relevant solutes, remain insufficiently characterized. In addition, the potential of this combination for reducing *L. monocytogenes* on food-contact surfaces has received limited attention.

In this context, the present study evaluated the combined effects of mild heat, RL, pH, and reduced a_w_ on the inactivation of *L. monocytogenes* ATCC 19,112. NaCl and sucrose were investigated as distinct solutes to determine whether the nature of the solute influenced the response to the combined treatment. The treatment was further evaluated on stainless-steel surfaces to provide an additional assessment under a food-processing-related condition. We hypothesized that combining these hurdles would enhance the inactivation of *L. monocytogenes* compared with the individual treatments and provide insight into the potential use of RL as part of a hurdle approach for microbial control.

## Materials and methods

### Microorganisms, culture media, and biosurfactant

*Listeria monocytogenes* ATCC 19112 stock cultures were maintained in Tryptone Soy Broth supplemented with 20% (v/v) glycerol and stored at -20 °C and - 80 °C.

The strain was cultivated in Tryptone Soy Broth or Agar (NeoGen^®^) supplemented with 6 g/L yeast extract (TSEYB and TSEYA, pH 7.2). For subsequent assays, the medium was modified with different concentrations of NaCl (0 to 15% w/v) and sucrose (0 to 50% w/v) to achieve distinct water activity (a_w_) levels. After sterilization, the pH of each medium was adjusted to values ranging from 5.0 to 7.0 using 0.5 M HCl or NaOH, followed by membrane filtration (0.22 μm).

Commercial rhamnolipid (Sigma-Aldrich^®^, R90) with 90% purity was used throughout the study. The RL stock solution was prepared in distilled water (50,000 mg/L), sterilized by filtration through a 0.22 μm membrane, and stored under refrigeration at 5 °C. Working solution was further prepared by tenfold dilution of the RL stock in distilled water.

### Water Activity (a_w_)

Water activity was determined using a LabStart-aw apparatus (Novasina, Switzerland) at room temperature (28.2 ± 1.4 °C). All measurements were performed in triplicate, and distilled water was used as the control.

### Combined effect of pH, solutes, and RL on the thermal treatment of *L. monocytogenes*

#### Inoculum preparation

Stock cultures of *L. monocytogenes* were streaked onto TSYEA plates and incubated at 37 °C for 24 h. After incubation, isolated colonies were transferred to fresh TSYEA plates and incubated again at 37 °C for 24 h. The resulting colonies were suspended in 5 mL of saline solution (0.86% w/v NaCl), centrifuged at 7,000 rpm for 5 min (MiniSpin Centrifuge, Eppendorf^®^), and the supernatant was discarded. The cell pellet was resuspended in 1 mL of saline solution and centrifuged again under the same conditions. After discarding the supernatant, the cells were suspended in 1 mL of TSYEB. The cell suspension was adjusted spectrophotometrically (Genesys 10-S, Thermo Fisher Scientific^®^) to an optical density (660 nm) of 0.8, corresponding to approximately 2 × 10^10^ CFU/ mL. This initial inoculum was then added to TSYEB prepared under different a_w_ and pH conditions.

#### Thermal treatment

Assays were performed in Eppendorf tubes containing 900 µL of the medium adjusted to specific pH and solute concentrations. Aliquots of 100 µL of the inoculum were added to the tubes, which were previously equilibrated at the test temperature. After inoculation, tubes were maintained at 62 °C for 10 min in a dry bath incubator (Kasvi). Non-heated samples were used as controls. Subsequently, the samples were subjected to serial dilutions, and the number of viable cells was determined by the drop-plate method (Miles et al. 1938) using 15 µL drops of each dilution. After incubation at 37 °C for 24 h, the plated drops containing 5–50 colonies were counted and result expressed as CFU/mL ( detection limit 3.33 × 10² CFU/mL).

#### Combined thermal and RL treatment

Thermal treatment was also conducted in the presence of RL (5,000 mg/L). Treatment with RL at room temperature was used as a control. The initial and final cell populations were compared among individual treatments (temperature or RL alone) and their respective combination under different a_w_ (solute concentrations) and pH values.

#### Expression of results

Treatment efficiency was expressed as the Log Reduction Factor (LRF), calculated as follows:

$$LRF\;=\;\log\;N_o\;-\;\log\;N$$

where N₀ is the initial cell count and N is the final cell count after treatment.

The occurrence of synergistic or additive effects between temperature and RL was evaluated by comparing the sum of individual effects (theoretical additive effect) with the inactivation achieved by the combined treatment, as described by Feyaerts et al. (2015).

### Scanning electron microscopy (SEM)

Structural changes in the cells induced by the treatments were visualized using scanning electron microscopy. Samples were prepared according to the methodology described by Bertuso et al. (2022). After gold sputter-coating, the specimens were observed under a JEOL FEG-SEM microscope (model JSM 7200 F) operating at 10 kV.

### Surface sanitization

Surface sanitization assays were adapted from Ivers et al. (2024). Briefly, AISI 304 stainless steel coupons (15 mm x 15 mm) were previously cleaned and sterilized by autoclaving. *L. monocytogenes* inocula were deposited onto the surface of each coupon as five 10 µL droplets. The inoculated coupons were left at room temperature for 90 min to allow complete drying of the droplets. The coupons were then transferred to test tubes containing 4 mL of distilled water adjusted to pH 5, in the presence or absence of NaCl (aw = 0.87) and rhamnolipid (5,000 ppm), and preheated to 62 °C. Following heat treatment at different time intervals, the tubes were vortexed at maximum speed for 30 s. The resulting suspensions were serially diluted in distilled water, and viable cells were enumerated using the drop plate method.

### Statistical analysis

For each independent experiment, three technical replicates were performed. Results are presented as the mean ± standard deviation of at least three independent experiments. Whenever appropriate, data were subjected to analysis of variance (ANOVA) followed by Tukey’s test at a 95% confidence level using OriginPro 2025 software (OriginLab Corporation^®^, USA). Statistically significant differences were indicated by different lowercase letters, whereas identical letters indicated the absence of significant differences among treatments.

## Results

*L. monocytogenes* ATCC 19112 was selected as the model strain because of its ability to survive under the pH and water activity (a_w_) conditions evaluated in this study. Preliminary experiments were conducted to determine the strain’s sensitivity to moderate heat treatment and to establish the experimental conditions. Based on these previous results, the treatment at 62 °C for 10 min was selected as the reference condition for subsequent experiments.

### Effect of combined treatments in presence of NaCl

**Fig. 1:**
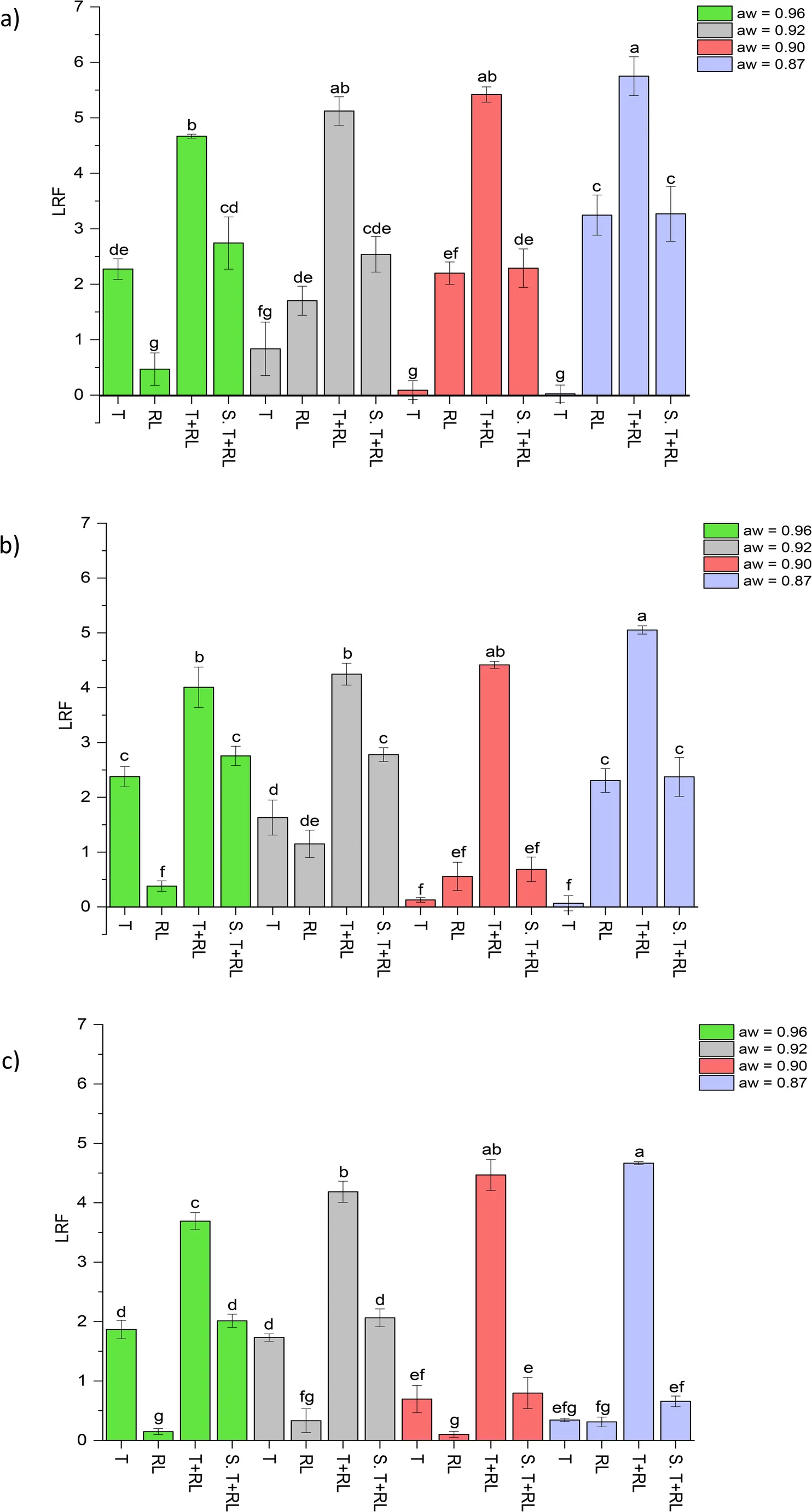
Log reduction factor (LRF) of *Listeria monocytogenes* viability after heat treatment (T), rhamnolipid (RL), the combined treatment (T + RL), and the sum of the individual effects (S.T + RL) in the presence of NaCl (a_w_ 0.96–0.87) at pH 5 (**a**), pH 6 (**b**), and pH 7 (**c**). Within each panel, bars followed by the same letter are not significantly different (*p* ≤ 0.05)

The survival of *L. monocytogenes* following heat treatment, rhamnolipids (RL) treatment, and the combined treatment (RL + heat) was evaluated under different a_w_ values obtained by progressively increasing the NaCl concentration in the culture medium. The influence of pH was assessed by adjusting the culture medium to each of the pH values evaluated. To determine whether the combined treatment exhibited synergistic activity, the corresponding log reductions were calculated for each experimental condition. The effects of the individual treatments, the combined treatment, and the theoretical sum of the individual effects are shown in Fig. 1.

It can be observed that increasing the solute concentration reduced the thermal inactivation of *L. monocytogenes*. At a_w_ 0.96 (without NaCl addition), heat treatment at 62 °C for 10 min resulted in approximately 2-log reduction in the bacterial population at all pH values tested. The addition of 5%, 10%, and 15% NaCl progressively reduced the efficacy of the heat treatment, particularly at a_w_ 0.90 and 0.87.

The antimicrobial activity of RL was also influenced by a_w_. However, unlike the effect observed for heat treatment, increasing the NaCl concentration enhanced RL activity under acidic conditions. By contrast, at pH 7.0, RL activity remained limited regardless of the a_w_ value tested (Fig. 1c).

The combined hurdle (heat + RL) was more effective than either treatment alone, particularly under acidic conditions and at lower a_w_ values, resulting in reductions greater than 5 log CFU/mL in the viable cell population. Under all conditions tested, including the medium without added solutes (a_w_ 0.96), the combination of heat/RL produced greater reductions in cell viability than the sum of the individual treatments. Thus, the results indicate a synergistic interaction between heat and RL, according to the criterion proposed by Feyaerts et al. (2015). The synergistic effect was consistently observed under all experimental conditions, demonstrating that the interaction was not restricted to media with reduced water activity.

The limited antimicrobial activity of RL at neutral pH and the reduced efficacy of heat treatment at high osmolarity highlight the influence of environmental conditions on the effectiveness of the individual treatments. Increasing the NaCl concentration decreased the efficacy of heat treatment while enhancing the antimicrobial activity of RL under acidic conditions. These opposing effects may have contributed to the synergistic interaction observed for the combined treatment.

### Effect of combined treatment in presence of sucrose

**Fig. 2:**
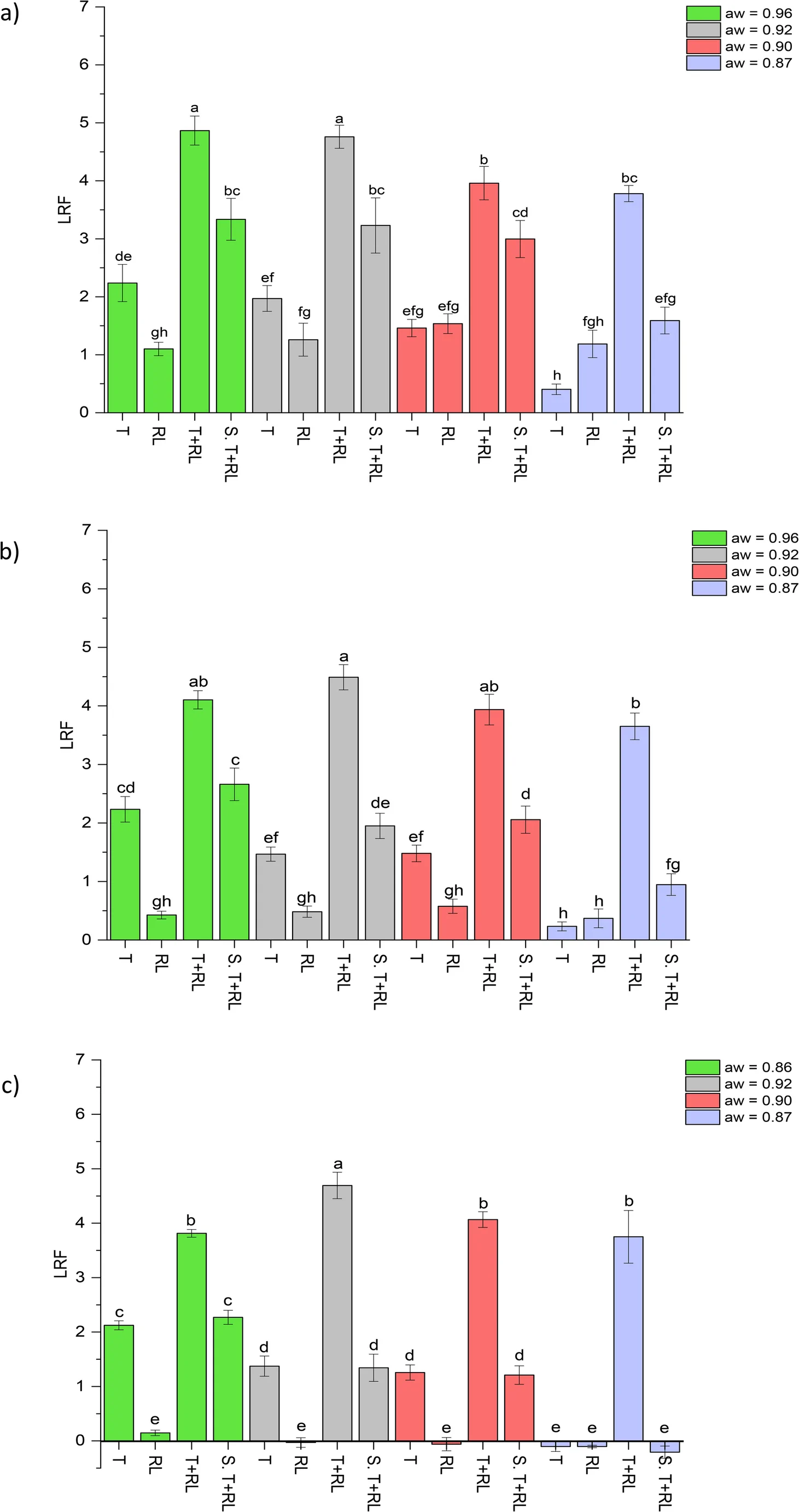
Log reduction factor (LRF) of *Listeria monocytogenes* viability after heat treatment (T), rhamnolipid (RL), the combined treatment (T + RL), and the sum of the individual effects (S T + RL) in the presence of sucrose (a_w_ 0.92–0.87) at pH 5 (**a**), pH 6 (**b**), and pH 7 (**c**). Within each panel, bars followed by the same letter are not significantly different (*p* ≤ 0.05)

To evaluate the influence of solute type on treatment efficacy, experiments were performed using sucrose, which, like NaCl, is a solute found in many food products. RL treatment was most effective at pH 5.0, resulting in an average reduction of approximately 1.5 log CFU/mL in viable cell counts (Fig. 2a). However, unlike the results obtained with NaCl, increasing the sucrose concentration in the culture medium did not affect RL activity, which remained comparable across all a_w_ values within the same pH range. Similarly to the observed in previous experiments, the combined hurdle exhibited synergistic activity at all pH and a_w_ values, reducing viable cell counts by approximately 4–5 log CFU/mL (Fig. 2).

Interestingly, under the lowest water activity (a_w_ = 0.87) at pH 7.0, the combined treatment produced the greatest reduction in *L. monocytogenes* viability (≅ 4 log), comparatively to the sum of treatments, which individually exhibited negligible effects (Fig. 2c). Similar behavior was observed with NaCl showing low LRF for individual treatments under such conditions (Fig. 1c). In contrast, at pH 5.0, the reduction achieved by the combined treatment was comparatively lower under both sucrose and NaCl (≅ 2.5 log) adjusted conditions (Figs. 1 and 2a).

### Scanning electron microscopy

**Fig. 3:**
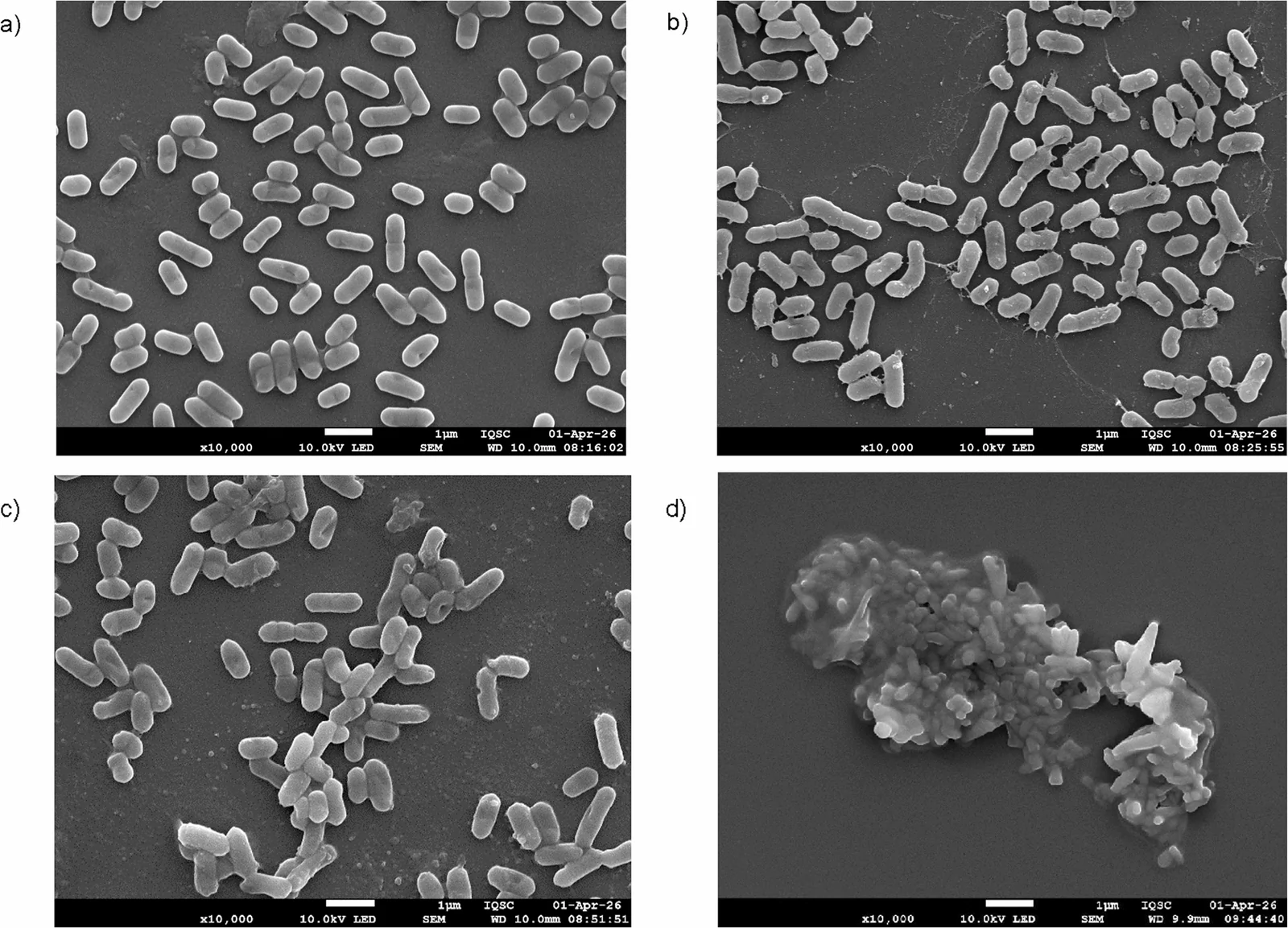
Scanning electron microscopy (SEM) images of *Listeria monocytogenes* cells in medium at pH 5.0 containing NaCl (a_w_ 0.87): (**a**) untreated control; (**b**) RL-treated cells (5000 mg/L); (**c**) heat-treated cells (62 °C for 10 min); and (**d**) cells subjected to the combined RL and heat treatment. Magnification: 10,000 X. The white scale bar represent 1 μm

The effects of heat and RL treatments on *L. monocytogenes* cells were further evaluated by scanning electron microscopy (Fig. 3). Bacterial cells were treated at pH 5.0 in the presence of NaCl (a_w_ = 0.87). Untreated cells exhibited the typical morphology of intact *L. monocytogenes*. Exposure to RL at room temperature for 10 min caused surface roughening and minor morphological alterations, including the appearance of filamentous structures (Fig. 3b). Heat treatment (62 °C for 10 min) induced cell collapse and deformation in a proportion of the population (Fig. 3c). By contrast, the combined treatment (RL + heat) resulted in only a few remaining cells, most of which were aggregated and displayed severe morphological damage compared with both the untreated control and the individual treatments (Fig. 3d).

### Surface sanitization test

To assess whether the results obtained in culture medium assays could be applied to the development of surface sanitization strategies, stainless steel coupons previously contaminated with *Listeria monocytogenes* were subjected to the different hurdles for varying contact times. In this test NaCl (a_w_ 0.87) was selected as model solute and the pH was adjusted to 5.

Exposure time markedly influenced the efficacy of the treatments (Table 1). At 10 and 5 min, heat treatment alone (T), heat combined with rhamnolipids (T + RL), and heat combined with rhamnolipids under low a_w_ (T + RL + NaCl) resulted in complete inactivation of *Listeria monocytogenes*, with no detectable bacterial growth. In contrast, heat treatment in the presence of NaCl (T + NaCl) produced LRF values of 1.06 and 0.31 after 10 and 5 min, respectively. Rhamnolipids applied without heat achieved LRF values of 1.20 and 1.02 in the presence of NaCl (RL + NaCl) and 1.09 and 0.83 in its absence after 10 and 5 min, respectively. When the exposure time was reduced to 1 min, none of the treatments completely inactivated *L. monocytogenes*. Heat treatment alone and its combination with RL resulted in similar reductions in cell population. However, the presence of salt in combination with RL (LRF = 1.00) or T + RL (LRF = 0.65) resulted in lower inactivation levels. The lowest reductions were observed for T + NaCl and RL treatments, which presented LRF values of 0.38 and 0.32, respectively.

**Table 1:** – Surface sanitization of stainless steel samples contaminated with *L. monocytogenes* using heat treatment (T, 62 °C for 10, 5, or 1 min), rhamnolipids (RL, 5000 mg/L), and their combination, in the presence or absence of NaCl (a_w_ = 0.87) at pH 5.0

Time (minutes)	Treatment	LRF ± SD
10	T + NaCl	1.06 ± 0.22
	T	*
	RL + NaCl	1.20 ± 0.13
	RL	1.09 ± 0.15
	T + RL + NaCl	*
	T + RL	*
5	T + NaCl	0.31 ± 0.12
	T	*
	RL + NaCl	1.02 ± 0.10
	RL	0.83 ± 0.17
	T + RL + NaCl	*
	T + RL	*
1	T + NaCl	0.38 ± 0.28
	T	1.82 ± 0.27
	RL + NaCl	1.00 ± 0.31
	RL	0.32 ± 0.17
	T + RL + NaCl	0.65 ± 0.23
	T + RL	1.81 ± 0.25

* below detection limit (< 2.52 log CFU/mL)

As previously observed, the presence of NaCl reduced the efficacy of heat treatment, whereas it enhanced the antimicrobial activity of RL. However, unlike the results obtained in culture medium, the surface sanitization assays showed that the presence of NaCl did not improve the inactivation of *L. monocytogenes* compared with heat treatment alone or heat combined with RL. Moreover, under the shortest contact time (1 min), heat treatment and heat/RL hurdle demonstrated similar reductions, indicating the absence of a synergistic effect. This finding is likely related to the limited contact time between the cells and the biosurfactant, as the antimicrobial efficacy of RL increased with longer exposure times (Table 1).

## Discussion

Heat causes the denaturation of enzymes, structural proteins, and nucleic acids, leading to damage to several cellular structures, including the cell wall, cytoplasmic membrane, and ribosomes (Russell 2003). Nevertheless, the extent of thermal damage depends on the temperature applied. At temperatures below 65 °C, disruption of the cytoplasmic membrane is considered the primary mechanism of thermal inactivation and is associated with increased membrane permeability and loss of membrane integrity (Teixeira et al. 1997). Heat-induced increases in membrane fluidity and permeability impair the regulation of transport processes, resulting in loss of cellular homeostasis under mild heating conditions and membrane rupture at higher temperatures (Cebrián et al. 2017).

The antimicrobial activity of RL is also primarily associated with the cytoplasmic membrane. Owing to their amphiphilic nature, RL molecules readily interact with membrane phospholipids, increasing membrane permeability and promoting leakage of intracellular metabolites, ultimately leading to cell lysis (Sotirova et al. 2008). Since both moderate heat treatment and RL compromise membrane integrity, the synergistic effect observed in the present study probably is associated with the simultaneous action of both treatments on the same cellular target.

The increased tolerance of microorganisms to heat treatment under reduced water activity has been extensively reported for several bacterial species, including *L. monocytogenes* (Fernandez et al. 2007). This phenomenon has been attributed to the reduced molecular mobility of water in highly concentrated solutions, which stabilizes proteins and ribosomes and consequently increases cellular resistance to thermal stress (Syamaladevi et al. 2016). Because RL are surface-active molecules that reduce both surface and interfacial tension, they may facilitate heat transfer or heat-induced membrane disruption under low-a_w_ conditions. In addition, RL self-assembly is temperature-dependent, and supramolecular structures such as micelles and vesicles undergo structural rearrangements as temperature increases (Ortiz et al. 2021). These changes may alter the interaction between RL molecules and the bacterial membrane, thereby contributing to the enhanced antimicrobial activity observed during the combined treatment.

Notably, synergism was also observed in the absence of added solutes (a_w_ 0.96). Under these conditions, heat treatment at 62 °C reduced viable cell counts by approximately 2 log CFU/mL, whereas the combined treatment achieved reductions exceeding 4 log CFU/mL. These findings indicate that high osmolarity, although protective against heat alone, is not essential for the synergistic interaction between heat and RL.

RL behave as anionic surfactants at neutral or alkaline pH but predominantly as nonionic surfactants under acidic conditions because of protonation of the carboxyl groups present in the hydrophilic moiety. The reported pKa values are 5.9 for mono-RL and 5.6 for di-RL (Ishigami et al. 1987; Abbasi et al. 2013). Below these pKa values, RL molecules remain predominantly protonated (COOH), minimizing electrostatic repulsion with the negatively charged bacterial membrane and facilitating membrane interaction (Ferreira et al. 2019). At neutral or alkaline pH, the carboxyl groups are predominantly deprotonated (COO−), resulting in anionic RL molecules.

In the presence of NaCl, electrostatic shielding of negatively charged polar groups by Na+ ions may reduce repulsive forces among RL molecules, and thereby favoring their interaction with the bacterial membrane (Helvaci et al. 2004). This mechanism may explain the enhanced antimicrobial activity observed at increasing NaCl concentrations, suggesting that NaCl plays a greater role in enhancing RL activity under conditions in which the biosurfactant is predominantly in its anionic form.

Similarly, the influence of pH on RL activity was also observed in the presence of sucrose. At a given water activity, the antimicrobial action of RL increased as the pH decreased (Fig. 2). Sucrose may interact with RL molecules through dipole–dipole interactions, hydrogen bonding, and hydrophobic interactions (Chauhan et al. 2016). However, unlike NaCl, increasing the sucrose concentration within each pH level did not enhance RL antimicrobial activity. This finding contrasts with a previous study by Passos and Nitschke (2024a ) and may be explained by the short treatment time (10 min), which may have been insufficient to reveal the effect of increasing sucrose concentration on RL activity. The influence of solute type on RL activity has also been reported by Lenaerts et al. (2023), who evaluated the susceptibility of *L. monocytogenes* Scott A to RL in buffer solutions with an a_w_ 0.92 adjusted with either NaCl or glycerol. RL exhibited significantly greater antimicrobial activity in the presence of NaCl, demonstrating that solute type is a key factor affecting RL efficacy. Overall, ionic solutes appear to enhance RL antimicrobial activity more effectively than nonionic solutes, consistent with the findings of the present study.

SEM observations were consistent with the microbiological results. Heat treatment alone produced limited changes in the bacterial population (Figs. 1a and 2a), whereas RL treatment was associated with a reduction in viable cells and alterations in cell surface morphology. SEM images alone do not allow direct confirmation of membrane damage; however, the morphological changes observed are consistent with the well-established membrane-disruptive activity of rhamnolipids, which can promote membrane destabilization, solubilization of membrane components, and leakage of intracellular material (Sotirova et al. 2008; Tonyali et al. 2019). Although RL alone reduced cell viability, complete inactivation was not achieved, probably because the 10 min exposure time was insufficient to eliminate the entire bacterial population. In contrast, the combined treatment resulted in reductions exceeding 5 log CFU/mL. The small number of cells observed by electron microscopy, together with the pronounced morphological damage, suggests substantial structural disruption. These observations are consistent with the hypothesis that the synergistic interaction involves the combined effects of heat and RL on bacterial cell integrity. Nevertheless, this interpretation remains speculative, and further studies are required to validate the underlying mechanism.

Heat treatment is widely used to control pathogenic and spoilage microorganisms in foods; however, it may also cause undesirable nutritional and sensory changes (Maurya et al. 2025). One strategy to minimize these adverse effects is the application of moderate temperatures for longer processing times (Greel 2024). Although moderate heat treatment alone may be insufficient to achieve the desired level of microbial inactivation, it can increase bacterial susceptibility to additional antimicrobial agents, thereby enhancing overall treatment efficacy when applied in combination (Barba et al. 2017). Moreover, combined preservation strategies may reduce the emergence of stress-tolerant cells by simultaneously targeting multiple cellular structures and processes, thereby limiting the effectiveness of adaptive stress responses (Karbowiak et al. 2023).

Sanitization assay does not show a synergistic interaction among the factors and further studies are needed to understand the limitations of the test as well as, the interaction of heat /RL with the cells adhered in the surface. Possibly, surface-attached cells behave differently from planktonic cells in suspension. Although they were not organized as biofilms, attached cells may experience a distinct microenvironment and specific chemical interactions with the material surface, which can influence treatment efficacy (Lavoie et al. 2025).

The applicability test provided preliminary evidence that the heat/RL combination can enhance antimicrobial efficacy through a hurdle-based approach. This strategy may offer an alternative to conventional chemical sanitizers, such as chlorine-based compounds, which raise environmental concerns. However, the stainless-steel coupon experiment was conducted under controlled laboratory conditions and does not fully reflect the complexity of food-processing environments, where factors such as the presence of organic matter, biofilms, surface characteristics, microbial strain, and the type of food-contact material may affect treatment efficacy (Arthur et al. 2025). Therefore, further studies under more representative conditions are needed to determine the practical effectiveness of the heat/RL combination as a sanitizing approach.

The integration of the hurdle concept with biosurfactant-based strategies remains largely unexplored, offering significant opportunities for future research. In particular, combining RL with physical preservation methods other than heat, especially for temperature-sensitive products, or with chemical hurdles, such as essential oils or other bioactive compounds, may expand their application and further enhance antimicrobial efficacy. As an example, a recent study demonstrated the potential of combining the bioactive phytochemical ungeremine with RL to develop a green sanitizing formulation for minimally processed foods. The washing of lettuce leaves with the RL–ungeremine formulation resulted in greater microbial reduction than the conventional sodium hypochlorite treatment. The formulation also exhibited strong antimicrobial activity against *Staphylococcus aureus*, *Escherichia coli*, and *Salmonella* Typhimurium. Furthermore, it preserved the visual quality of lettuce leaves for up to eight days and showed no significant ecotoxicity (Caraturro et al. 2025).

The combination of RL and moderate heat enhanced the inactivation of *L. monocytogenes* across the pH and water activity conditions evaluated, suggesting the potential of this hurdle-based approach for pathogen control under the tested conditions. The observed antimicrobial effects may also be relevant to high-solute food environments, such as brines and syrups, where conventional sanitization strategies may be less effective; however, the extent to which these findings apply to actual food matrices remains to be validated. Further studies under more representative food-processing conditions and involving multiple *L. monocytogenes* strains are needed to assess the consistency of the observed effects and the broader applicability of the findings.

## Conclusions

The synergistic interaction between rhamnolipids and moderate heat observed in high-solute conditions highlights the potential of this hurdle-based approach for controlling *Listeria monocytogenes* under the conditions evaluated. Furthermore, the integration of rhamnolipids with physical preservation methods may represent an alternative strategy for enhancing microbial control in the food industry.

## Data Availability

Data will be made available under request
